# Hetero-Element-Doped Molybdenum Oxide Materials for Energy Storage Systems

**DOI:** 10.3390/nano11123302

**Published:** 2021-12-06

**Authors:** Bo Hu, Shuofeng Jian, Ge Yin, Wenhao Feng, Yaowen Cao, Jiaxuan Bai, Yanan Lai, Huiyun Tan, Yifan Dong

**Affiliations:** 1 Engineering Research Center of Nano-Geomaterials of Ministry of Education, Faculty of Material Science and Chemistry, China University of Geosciences, Wuhan 430074, China; hubocug@cug.edu.cn (B.H.); jianshuofeng@163.com (S.J.); yinge3111818157@126.com (G.Y.); wenhaofeng5@gmail.com (W.F.); caoyaowen12@163.com (Y.C.); Phoenix9360@yeah.net (J.B.); nannan2002617@163.com (Y.L.); 2 Faculty of Material Science and Chemistry, China University of Geosciences, Wuhan 430074, China

**Keywords:** molybdenum oxides, hetero-element doping, energy storage materials

## Abstract

In order to meet the growing demand for the electronics market, many new materials have been studied to replace traditional electrode materials for energy storage systems. Molybdenum oxide materials are electrode materials with higher theoretical capacity than graphene, which was originally used as anode electrodes for lithium-ion batteries. In subsequent studies, they have a wider application in the field of energy storage, such as being used as cathodes or anodes for other ion batteries (sodium-ion batteries, potassium-ion batteries, etc.), and electrode materials for supercapacitors. However, molybdenum oxide materials have serious volume expansion concerns and irreversible capacity dropping during the cycles. To solve these problems, doping with different elements has become a suitable option, being an effective method that can change the crystal structure of the materials and improve the performances. Therefore, there are many research studies on metal element doping or non-metal doping molybdenum oxides. This paper summarizes the recent research on the application of hetero-element-doped molybdenum oxides in the field of energy storage, and it also provides some brief analysis and insights.

## 1. Introduction

The rapid growth of the population and industrial production have put great pressure on natural resources, and, with the depletion of fossil energy and the rapid development of electronic products, the demands for high energy density and power density energy storage equipment, such as ion batteries and supercapacitors, continues to grow [1,2,3]. Over the last few decades, lithium-ion batteries (LIBs) have become the favorite in the consumer electronics market due to their good energy density, long cycle life, and environmental friendliness. The abundant mineral materials in nature are used as the matrix for the research of electrode materials for lithium-ion batteries [4]. As we know, transition metal oxides are appropriate for the insertion/extraction of small ions, such as H^+^ and Li^+^, so they are candidates for a wide range of applications, including energy storage devices [5]. Among the transition metal oxides, molybdenum oxides have diverse crystal structures, oxidation states, and particular physicochemical properties, such as conductivity, mechanical and thermal stability, as well as cyclability [6]. In addition, they are wide band gap n-type semiconductors, which plays a vital role in technological applications [7]. At the same time, graphite materials have been successfully marketed as anodes for LIBs for decades, but the low theoretical specific capacity of 372 mA h g^−1^ has gradually failed to meet the growing demand for high energy density batteries [8,9]. Molybdenum oxide, particularly MoO_2_, has received considerable attention due to its metallic conductivity and high theoretical capacity (838 mA h g^−1^) [10]. Therefore, molybdenum oxides, as cathode or anode materials, appear to be promising candidates for ion secondary batteries and supercapacitors.

There has been a great deal of research on pure molybdenum oxides using different methods, such as thermal decomposition [11], chemical vapor deposition [12], hydrothermal-solvothermal process [13,14,15], thermal evaporation, and nanocasting, to synthesize nanostructure molybdenum oxides to increase electrochemical performance. However, the interlamellar spacing of pure molybdenum oxides is limited, it is difficult to enhance their specific capacities, and they cannot hold big ions, such as Na^+^, K^+^, Zn^2+^, and Mg^2+^. Therefore, it is necessary to approach them from another perspective to improve the electrochemical performance of molybdenum oxide-based materials. Doping with different elements is an effective strategy, which can increase the distance between the layers in the crystal structure, change the layered crystal structure to a tunnel-type structure, or enhance conductivity.

**Figure 1:**
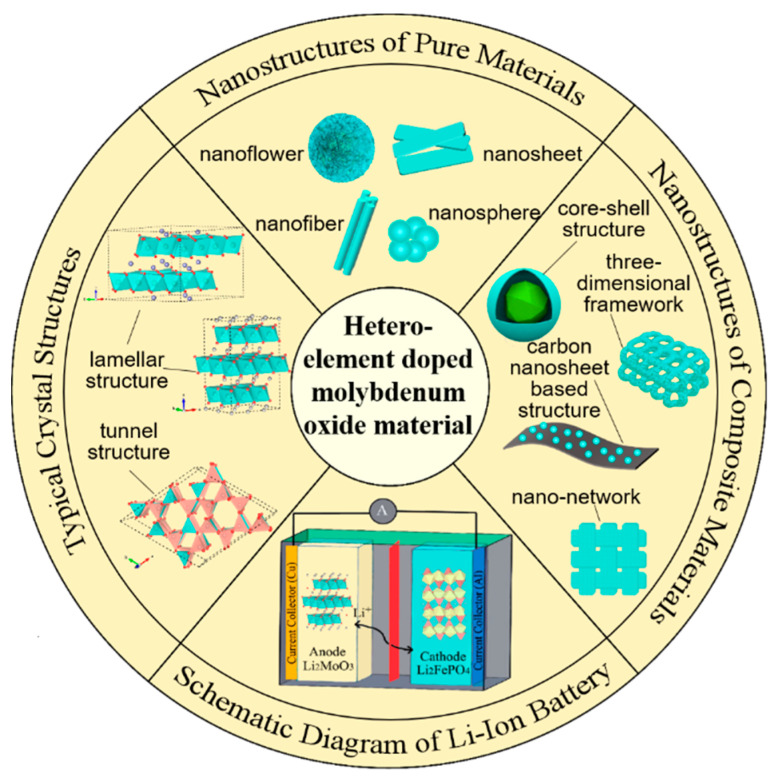
Summarization of some typical crystal structures and nanostructures of hetero-element-doped molybdenum oxide materials, as well as the schematic diagram of their application in energy storage systems.

Hetero-element doping molybdenum oxides can be divided into several varieties, namely alkali metal (Li, Na, or K) and alkaline earth metal (Mg or Ca) doping, transition metal (Zn, Ni, Mn, Ti, or Co) or their oxides doping, non-metal (C or N) doping, as well as non-metal-/metal-doped composites. Metal doping molybdenum oxides are attractive as hosts for ions, which can form multiple crystal structures or phases. The combination of dissimilar metal with molybdenum oxides is able to produce materials with new architectural and chemical characteristics; thus, a variety of systems could be explored [6]. In addition, some metal-doped molybdenum oxides have a pseudocapacitive property according to the literature [16,17]. Non-metal doping is a common way of material modification, while the frame structure composed of carbon or nitrogen can greatly increase the specific surface area of the materials and enhance conductivity. Because molybdenum has different oxidation states in the compounds, molybdenum oxides doped with different elements have a variety of chemical compositions and crystal phases, and there are bound to be many synthetic methods to obtain new materials. In this review, we primarily highlight and summarize the variety of hetero-element-doped molybdenum oxide materials and their synthetic methods, as well as their electrochemical performance as energy storage materials. Figure 1 summarizes some typical crystal structures and nanostructures of hetero-element-doped molybdenum oxide materials, as well as the schematic diagram of their application in energy storage systems; the details will be introduced later.

## 2. Alkali Metal and Alkaline Earth Metal-Doped

### 2.1. Alkali Metal-Doped

**Figure 2:**
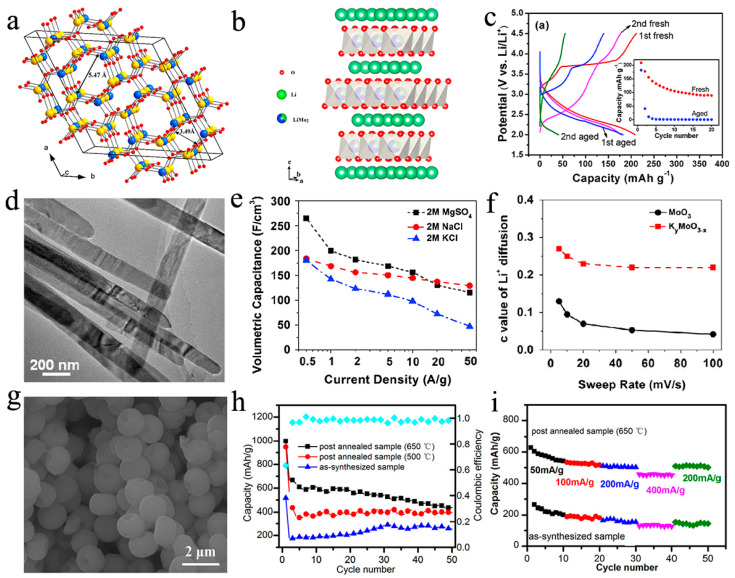
(**a**) Crystal structure of the rhombohedral modification of Li_2_MoO_4_. Blue and yellow spheres represent Mo and Li ions, respectively. Narrow and wide channels along the c-axis feature 4-member rings and 6-member rings, respectively. Reproduced with permission. [30] Copyright 2015, American Chemical Society. (**b**) Lattice structure of Li_2_MoO_3_. (**c**) Charge and discharge potential curves of the fresh and aged Li_2_MoO_3_; the inset is for their cycling performances. Reproduced with permission. [31] Copyright 2014, Elsevier B. V. (**d**) Low-resolution TEM image of K_y_MoO_3−x_ nanobelts. (**e**) Volumetric capacitance and current density for K_y_MoO_3-x_ in different electrolytes. (**f**) Numbers of Li^+^ inserted in the tunneled MoO_3_ and K_y_MoO_3−x_ (c value of Li^+^ diffusion). Reproduced with permission. [16] Copyright 2015, Elsevier B. V. (**g**) Morphological of as-synthesized CaMoO_4_ microspheres by reaction time of 0.5 h. (**h**) Cycling performance of the CaMoO_4_ microspheres before and after being heat-treated at different temperatures at current densities of 200 mA g^−1^. (**i**) Rate performance of CaMoO_4_ microspheres before and after calcination at 650 °C. Reproduced with permission. [32] Copyright 2018, Springer Nature.

Alkali metal-doped molybdenum oxides mainly include A_x_MoO_2_, A_x_MoO_3_, A_2_MoO_4_ (A = Li, Na, or K), Li_4_Mo_3_O_8_, and Li_2_Mo_4_O_13_ [18,19,20,21,22,23,24]. Most LiMO_2_ (M = Co, Mn, Ni, Fe) is associated with rock-salt architectures and is an ordered or distorted form of NaCl. AMoO_2_ (A = Li, Na or K) is isostructural to well-known LiMO_2_ materials for LIBs, having the potential to be the lithium storage material. Per unit of MoO_2_ can hold up to 0.85 Li at an average potential of 3 V [19,25]. Li_2_MoO_3_ has a novel, disordered *α*-NaFeO_2_ structure (R-3m) with the molybdenum present as Mo_3_O_13_ clusters (Figure 2b), firstly determined by James and Goodenough [26]. The Mo^4+^ in the Li_2_MoO_3_ compound can be oxidized to Mo^6+^ oxidation state when the Li_2_MoO_3_ is charged to 4.4 V (vs. Li^+^/Li). Therefore, Li_2_MoO_3_ can carry out a two-electron electrochemical reaction per unit molecule, so it has a huge potential for energy storage (theoretical capacity is about 339 mA h g^−1^) [27,28]. The crystal of Li_2_MoO_4_ (Figure 2a) shows a tunnel structure, consisting of a three-dimensional network of corner-linked, slightly distorted LiO_4_ and MoO_4_ tetrahedra along crystallographic c-axis [29,30]. As is well-known, materials with NASICON-type structure are also promising candidates for energy storage systems.

Recently, many studies about alkali metal-doped molybdenum oxides have been reported. Ma et al. [31] investigated the structural and performance stability of Li_2_MoO_3_ in air. They found that Li_2_MoO_3_ can be oxidized/decomposed in air at room temperature. The oxidation of Mo^4+^ and the consumption of Li^+^ led to the generation of Li^+^ vacancies, Mo^4+^ migration, Li-Mo inversion, and the destruction of Mo_3_O_13_ clusters in Li_2_MoO_3_. Therefore, as shown in Figure 2c, the aged material (storing the phase-pure Li_2_MoO_3_ in desiccators with a relative humidity of below 10% at room temperature for 120 days) delivered more attenuation of capacity after the initial cycle. Verma and coworkers [24] devised a novel procedure for the synthesis of Li_2_Mo_4_O_13_ using a single-step conventional solid-state reaction. The rod-like (size range of 1–10 μm) material shows an initial reversible capacity of 1062 mA h g^−1^ at 0.1 C (up to theoretical capacity), and the capacity retention is 74% over 50 cycles with coulombic efficiency close to 98% during the subsequent cycles, proving that Li_2_Mo_4_O_13_ electrodes exhibit high reversible capacity and great cycling performance. K^+^ pre-intercalated hydrogenated MoO_3_ (K_y_MoO_3−x_) material was reported by Xiao [16]. The MoO_3_ nanobelts were synthesized using the hydrothermal method, hydrogenation was used to introduce oxygen vacancies into MoO_3_ nanobelts (Figure 2d), and the resulting MoO_3−δ_ was further reacted with KBH_4_. It was proved that several kinds of ions (Li^+^, Na^+^, K^+^, and Mg^2+^) with different ion dimensions can embed into the K_y_MoO_3−x_. In Figure 2e,f, the authors calculated volumetric capacitance at different current density for K_y_MoO_3−x_ in three electrolytes, as well as the number of inserted Li^+^ in K_y_MoO_3−x_ and MoO_3_. It is noted that Mg^2+^ has the greatest capacitance, while Na^+^ has the best retaining capacitance, and, when the sweep frequency is augmented to 100 mV s^−1^, the quantum of inserted Li^+^ in K_y_MoO_3−x_ remains almost invariant (from 0. 27 to 0.22), but only 0.042 Li^+^ is embedded in MoO_3_, demonstrating that K_y_MoO_3−x_ has a wider interlayer distance and stronger conductivity compared to pure MoO_3_. Zhu et al. [33] reported a novel layered sodium molybdenum oxide Na_0.3_MoO_2_ synthesized by solid-state reaction as an anode material for sodium-ion batteries (SIBs). The crystal and nanostructure of Na_0.3_MoO_2_ are both layered morphologies, and the long-term cycling performance shows an excellent capacity retention of 72.4% after 1000 cycles, and the coulombic efficiency is up to about 100%, except for the preliminary cycle.

In addition to single element doping, there are also some double elements-doped materials. In the existing research, potassium is doped in Li-rich molybdenum-based oxide or sodium molybdate. Yu et al. [34] prepared K-doped Li_2_MoO_3_ cathode material for the first time; the K-doped MoO_3_ nanobelts were synthesized by hydrothermal method and used as templates and then annealed at high ambient temperatures. The larger K^+^ could not be extracted from the interlayer during de-lithiation, staying in the Li slab to restrain the phase transformation, so the doped samples are more stable. The obtained Li_1.9_K_0.1_MoO_3_ has a satisfying initial capacity (235 mA h g^−1^ at 0.05 C rate, 1C = 320 mA g^−1^). Additionally, after a certain number of cycles, it maintains 56% of its initial capacity, which is much better than the undoped sample Li_2_MoO_3_ (38%). The enhanced electrochemical performance may be due to the change in the valence state of Mo^4+^ in the Li_1.9_K_0.1_MoO_3_ crystal, making the sample hardly release oxygen even if it is charged to a potential of 4.4 V (vs. Li^+^/Li). Serdtsev and coworkers [35] predicted the possible sodium-ion pathways and the energy barriers for sodium diffusion in the glaserite-type A_3−x_Na_1+x_(MoO_4_)_2_ (A = Cs, K) through the approximate bond valence sum approach. The double molybdate K_3_Na(MoO_4_)_2_ has a monoclinic distorted superstructure (space group C2/c) with a quadruple unit cell, which corresponds to a much lower energy barrier for migration through the K site compared to the normal sodium site, while the ionic conductivity of sodium can be strengthened for pyrochlore doped with divalent or trivalent ions. At present, there are relatively few studies related to bimetallic elements-doped molybdenum oxide materials. However, according to the existing work, it can be seen that this strategy is expected to obtain electrode materials with a long cycle life and further improve the performance of single metal-element-doped molybdenum oxide materials.

### 2.2. Alkaline Earth Metal-Doped

Ca_x_MoO_3_, CaMoO_4_, Mg_x_MoO_3_, and MgMoO_4_ [36,37,38,39] are several common alkaline earth metal doping molybdenum oxides. CaMoO_4_ adopts a tetragonal scheelite structure with the Mo in tetrahedral O-coordination [37]. The CaMoO_4_ microspheres with nano-pits were synthesized through the facile hydrothermal method by You et al. [32]. The SEM of CaMoO_4_ microspheres is shown in Figure 2g; the nano-pits distributed on the CaMoO_4_ microspheres can alleviate the volume expansion problem and increase the specific surface area and wettability with the electrolyte, thus improving cycle stability and electrochemical performance. They discovered that the discharge capacity of CaMoO_4_ tends to increase with increasing calcination temperature. In Figure 2h,i, for CaMoO_4_, which is annealed at 650 °C, the leftover organic matter is fully carbonized and the best crystallinity is obtained after the high temperature treatment. When the post-annealed CaMoO_4_ is used as anode for Li-ion battery, it has a higher reversible capacity (434 mA h g^−1^ at 200 mA g^−1^ after 50 cycles) than the theoretical capacity of graphite (372 mA h g^−1^). The single crystals of MgMoO_4_ displayed monoclinic symmetry and adopted the C2/m space group at normal temperature and pressure [40]. Haetge [41] reported the synthesis of templates for porous MgMoO_4_ films with nanocrystalline walls. These materials were produced by solution phase co-assembly of chlorinated salt precursors with KLE-type poly(ethylene-co-butylene)-block-poly (ethylene oxide) di-block copolymers using the evaporation-induced self-assembly process. As the electrodes for the Li-ion battery, MgMoO_4_ thin films deliver 162 mA h g^−1^ at C/1.5 (1C is defined as the intercalation of 1 Li^+^ per formula unit in 1 h at 25 °C) after 100 cycles, and the capacity decreases only slightly between the 20th and 100th cycles. At the same time, the material employed in this work can be reversibly cycled at C/2 (175 mA h g^−1^), 1 C (167 mA h g^−1^), 2 C (147 mA h g^−1^), 4 C (130 mA h g^−1^), and 8 C (116 mA h g^−1^) rates. It can be known that not all alkaline earth metal-doped molybdenum oxide materials can have a high capacity, although their cycling stability is relatively excellent. This may be related to the crystal structure of the material itself, which determines the material’s ability to hold active ions. Of course, this is also reflected in other hetero-element-doped molybdenum oxide materials.

## 3. Transition Metal-Doped

**Figure 3:**
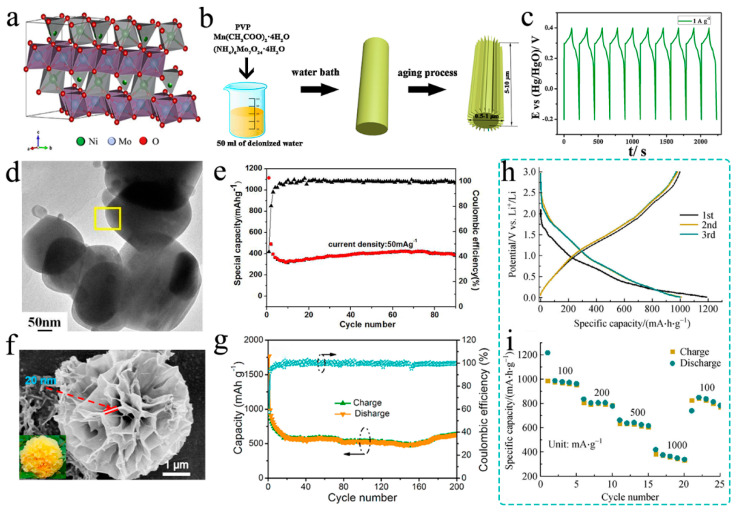
(**a**) Crystal structure of monoclinic NiMoO_4_ illustrating the coordination relationship of NiO_6_ and MoO_6_ octahedra. Reproduced with permission. [45] Copyright 2018, Elsevier Ltd. (**b**) Schematic diagram illustrating the formation process of rod-like MnMoO_4_ crystalline with two-dimensional nanoflakes. (**c**) GCD (galvanostatic charge–discharge) curves of the energy storage made from rod-like MnMoO_4_ crystalline in last 10 cycles at 1A g^−1^. Reproduced with permission. [46] Copyright 2017, Elsevier Ltd. (**d**) SEM image of α-ZnMoO_4_. (**e**) Cycling performance of α-ZnMoO_4_. Reproduced with permission. [47] Copyright 2017, Elsevier B. V. (**f**) SEM image of Cu_3_Mo_2_O_9_. (**g**) Cycling performance and coulombic efficiency of Cu_3_Mo_2_O_9_ at 0.1 A g^−1^. Reproduced with permission. [48] Copyright 2017, Elsevier B. V. (**h**,**i**) Discharge–charge voltage profiles and rate performance of Fe_2_Mo_3_O_8_ hollow spheres. Reproduced with permission. [49] Copyright 2020, Higher Education Press.

Two main forms of transition metal-doped molybdenum oxides exist, namely A_2_Mo_3_O_8_ and AMoO_4_ (A = transition metal) [42,43,44]. AMoO_4_ (A = transition metal), namely Mo-containing mixed oxides, are appropriate for ions to insertion, which can form multiple crystal structures or phases. The combination of dissimilar metal with molybdenum oxides is able to produce materials with new structures and chemical properties. The crystal structure of NiMoO_4_ belongs to monoclinic crystal system (Figure 3a), and the rod-like NiMoO_4_ produced through solution combustion synthesis was used for sodium batteries for the first time by Minakshi and coworkers [45], exhibiting a safe operation potential (~1.0 V vs. Na^+^/Na). The rod crystalline phasing consists of tiny NiO grains (30 nm clusters of 10 nm grains) solidly fixed to the NiMoO_4_ rods. The percentage of NiO rises with the quantity of oxidant, while the rods show cracks and fractures at the highest oxidant levels. These rods display a staggered morphology, allowing good surface pathways for ion exchange. Therefore, this hetero-structure material produces a high initial discharge capacity of 550 mA h g^−1^ at a current density of 0.05 A g^−1^. In addition, it has good capacity retention of 82% after 50 cycles. Rod-shaped MnMoO_4_ crystals with two-dimensional nanosheets have been satisfactorily produced by a new and facile wet chemical method with the help of polyvinylpyrrolidone by Liu’s group [46]. Figure 3b shows the schematic diagram of the synthesis process. The rod-shaped MnMoO_4_ is used as the work electrode for the supercapacitor and shows excellent long cycling and rate performance due to the two-dimensional nanosheets providing increased interfacial contact between the MnMoO_4_ active material and the electrolyte, which facilitates the diffusion and charge transfer of the electrolyte. Figure 3c further illustrates the charge/discharge curves at the current density of 1 A g^−1^ for last 10 cycles (totally cycled for 1000 cycles), and the coulombic efficiency remains nearly 100% for each cycle, with a completely reversible lithium storage process. Fei et al. [47] analyzed the electrochemical properties of α-ZnMoO_4_ nanoparticles as anode material for Li-ion battery by a simple co-precipitation method and subsequently calcined for the first time. TEM shows the morphological property of α-ZnMoO_4_ in Figure 3d, and the samples show a dispersed and uniform morphology with a diameter of 200–300 nm. As shown in Figure 3e, the as-prepared α-ZnMoO_4_ exhibits excellent electrochemical performance, including a very stable capacity of 389 mA h g^−1^ at 50 mA g^−1^ with capacity loss of 0.2% per cycle for the 80th cycle. It also has excellent rate performance, and the capacity is basically restored when the current density returns to the initial value.

LiHoMo_3_O_8_ was prepared successfully through carbothermal reaction at 750 °C by Das et al. [50]. The Li-storage and recyclability have been examined, which delivered a high level of capacity (~470 mA h g^−1^ at 30 mA g^−1^ in rate performance) under ambient temperature. This work lays the foundation for the study and understanding of the lithium cycling mechanism of molybdenum cluster oxides and their potential applications in lithium storage devices. In the meantime, there have been many studies of isostructural Mo-cluster mixtures in which Ho is substituted by Y and possibly other heavy rare earth ions, or those in which the Li and Ho ions are substituted by two divalent metal ions, such as transition metals [42,51]. Thus, more research on the use of A_2_Mo_3_O_8_ (A = transition metal) for energy storage has been reported [42,49,52,53]. Chowdari’s group [42] reported the electrochemical performance and the Li-cycling mechanism of Mn_2_Mo_3_O_8_ early. The synthesized Mn_2_Mo_3_O_8_ belongs to the P63mc space group and the Rietveld refined lattice parameters are: a = 5.795(2) Å and c = 10.254(2) Å. The first discharge capacity of Mn_2_Mo_3_O_8_ is about 710 mA h g^−1^, while it shows bad cycling performance. Recently, Zhang and coworkers [49] constructed novel self-assembled iron (II) molybdenum (IV) oxide hollow globules with a wall of 100 nm by a bubble-template-assisted hydrophile method of synthesis combined with simple calcination. The tight integration of miniature nanoparticles on the surface of the hollow globules not only offers more active sites for Fe_2_Mo_3_O_8_ but also contributes to the stability of the hollow structure, thus enhancing the lithium storage performance. The discharge–charge voltage profiles and rate performance of Fe_2_Mo_3_O_8_ hollow spheres are shown in Figure 3h,i; Fe_2_Mo_3_O_8_ hollow spheres deliver the initial discharge and charge capacities of 1189 and 997 mA h g^−1^ at 100 mA g^–1^, respectively, which are higher than the theoretical capacity of 813 mA h g^−1^ based on lithium storage mechanism of redox conversion. Additionally, in the rete performance, when the current rate decreases to 0.1 A g^–1^, a high discharge capacity of 812 mA h g^–1^ can still be regained, suggesting the desirable tolerance of the Fe_2_Mo_3_O_8_ electrode. Therefore, it is very important to improve the cycling stability and rate performance of the electrode materials by seeking suitable synthesis methods and modification methods.

In addition to these two types of transition metal-doped molybdenum oxides, A_2_Mo_3_O_8_ and AMoO_4_ (A = transition metal), Cu_3_Mo_2_O_9_ used as anode for Li-ion battery was reported by Guo’s group [48]. They developed a facile strategy to fabricate the 3D hierarchical Cu_3_Mo_2_O_9_ flowers via simply calcining the Cu_3_(OH)_2_(MoO_4_)_2_ nano-cuboids, which can be accomplished easily by one-pot hydrothermal method. The final hierarchical flower-like Cu_3_Mo_2_O_9_ was transferred from self-assembled rectangular parallelepiped single nanocrystal via calcination in air (SEM is shown in Figure 3f). They investigated the influence of temperature during the calcination, finding that the 3D hierarchical morphology of the Cu_3_Mo_2_O_9_ annealed at 600 °C is different either from the one that is composited of irregular nanoparticles at 400 °C or from the one that is excessively aggregated at 700 °C, which is the best in these three materials. As shown in Figure 3g, after 200 cycles, the Cu_3_Mo_2_O_9_ electrode was still capable of delivering a specific capacity of 632 mA h g^−1^ at 0.1 A g^−1^ with a large coulombic efficiency (~99%), demonstrating its remarkable cycling stability and reversibility. When designing experiments, we often need to set concentration gradients, temperature gradients, etc., to find the best reaction conditions. In fact, this is a very basic understanding for the synthesis of nanomaterials.

## 4. Non-Metal-Doped

Doping with non-metallic elements, such as carbon and nitrogen, is a typical method of electrode material modification. Carbon and nitrogen doping modification generally does not affect the crystal structure of molybdenum-based materials, but different synthesis methods, such as co-precipitation, hydrothermal technology, etc., can be used to obtain composite materials with different nanostructures. Although some molybdenum oxides possess metallic conductivity, it is necessary to improve on their electrical conductivity for better electrochemical performances. In addition, substantial volume expansion during repetitive cycling remains the key to limiting its practical deployment in lithium-ion batteries [54]. Crossbreeding with carbonaceous substrates, such as graphene, carbon nanotubes and meso/microporous carbon, carbon black, and carbon dots [55,56,57,58,59,60,61], is an efficient tactic to address these issues. The nanostructure of the composite material can increase the specific surface area of the material to a certain extent so that the material and the electrolyte can be more fully contacted. For doping methods, in situ synthesis, hydrothermal, calcination, etc., have been proven to be the simple and scalable approach [56,61,62]. Lu’s group [60] investigated the carbon vacancy defects using density functional theory (DFT); C vacancy formation in a perfect graphene matrix was taken as a reference. When one C atom is replaced by N, the relative C vacancy formation energy becomes more negative, indicating that C atoms are gradually easier to detach, especially when replaced by bonding to N (−2.99 eV), and N doping can effectively induce C defect formation. This kind of ability could improve the electron conductivity of electrodes and increase the active sites for ions storage. Therefore, besides doping carbon, additionally doping with nitrogen is also an effective strategy to modify materials.

**Figure 4:**
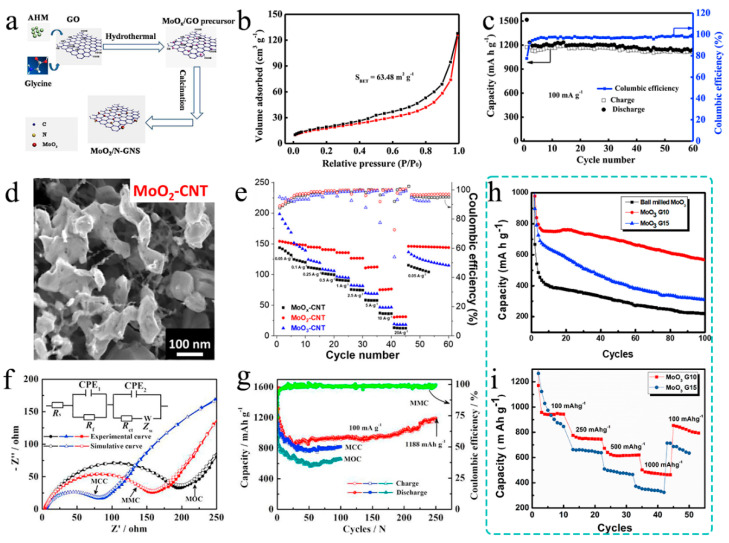
(**a**) Schematic illustration of the synthesis procedure of MoO_2_/N-GNS hybrid. (**b**) N_2_ adsorption–desorption isotherm. (**c**) Capacity vs. cycle number and the corresponding coulombic efficiency of MoO_2_/N-GNS hybrid electrode. Reproduced with permission. [63] Copyright 2014, Elsevier B. V. (**d**) SEM of MoO_2_-CNT after annealing at 500 °C in an argon atmosphere. (**e**) Specific de-lithiation capacity and coulombic efficiency derived from galvanostatic cycling at specific currents between 0.05 and 20 A·g^−1^. Reproduced with permission. [64] Copyright 2018, American Chemical Society. (**f**) The pore size distributions of MOC, MMC, and MCC samples. (**g**) The discharge–charge of MOC, MMC, and MCC samples at the current density of 100 mA g^−1^. Reproduced with permission. [65] Copyright 2017, Elsevier B. V. (**h**) Galvanostatic tests of ball-milled MoO_3_, MoO_3_-10 wt % rGO, and MoO_3_-15 wt % rGO. (**i**) Rate performance of MoO_3_-10 wt % rGO and MoO_3_-15 wt % rGO. Reproduced with permission. [66] Copyright 2019, Elsevier B.V.

In specific studies, Cao’s group [63] presented a two-step hydrothermal-calcination method to produce MoO_2_/N-doped graphene (MoO_2_/N-GNS) hybrids, in which MoO_2_ nanoparticles were homogeneously distributed on N-GNS sheets through Mo-N chemical bonds formed between MoO_2_ and N-GNS (Figure 4a). Benefiting from the substrate of graphene and N-doping, the mixture has a large specific surface area (Figure 4b). As Figure 4c indicates, this material was used as an anode for Li-ion battery, delivering a high initial discharge capacity of 1517 mA h g^−1^, and the MoO_2_/N-GNS still offered a reversible capacity of up to 1139 mA h g^−1^ at 100 mA g^−1^ after 60 cycles. Three molybdenum oxide-carbon nanotube hybrid materials were investigated as the negative electrodes in an Li-ion capacitor (LICs) by Fleischmann and coworkers [64] for the first time. They compared the properties of materials with different crystal structures and morphologies obtained by annealing after deposition. The SEM of MoO_2_-CNT (the best performance) is shown in Figure 4d, and flake crystals were observed in CNT networks with transverse dimensions up to 150 nm and thicknesses up to about 50 nm. Based on its excellent morphological characteristics, MoO_2_-CNT negative electrodes exhibit a superior rate handling, delivering a higher maximum capacity of around 150 mA h g^−1^ compared with MoO_x_-CNT and MoO_3_-CNT when the current density decreased to 0.05 A g^−1^ (Figure 4e). Additionally, the calculated high power density refers to 70 Wh kg^−1^ at 83 W kg^−^^1^. In fact, in molybdenum oxides, molybdenum dioxide itself has a pseudocapacitive property, so it is a matter of course that MoO_2_-CNT has the best performance as the electrode for Li-ion capacitor in these three materials. This also lets us know that, sometimes, we can make assumptions about the test results based on whether the matrix material has certain properties.

For an in situ synthesis method, Chai’s group [65] reported MoO_2_-Mo_2_C-C microspheres (MMC) synthesized via in situ carbonization as anode materials for lithium-ion batteries. Figure 4f shows the electrochemical impedance spectroscopy (EIS) of the materials. Obviously, carbon doping could reduce the charge transfer resistance, leading to a better electron conductivity, and ultimately enhance the diffusion of lithium ions to the MMC samples. Owing to the combination of the higher conductivity of Mo_2_C, C and the appropriate content of Mo_2_C, the MMC microspheres exhibit excellent cyclability and good stability. As shown in Figure 4g, for the MMC electrode, the initial discharge/charge specific capacities are nearly 1594/1171 mA h g^−1^ at 100 mA g^−1^, respectively, with a relatively low coulombic efficiency of 73.5%. The loss capacity was accorded with the irreversible procedure, including the insertion of Li^+^ into the MoO_2_ lattice and the formation of solid electrolyte interface (SEI) film. However, this material visibly delivers an excellent discharge capacity of 1188 mA h g^−1^ after 250 cycles. Recently, Li et al. [67] produced extremely small and widely dispersed MoO_x_ nanoparticles anchored on N-doped three-dimensional delaminated porous carbon (3D-MoO_x_@CN) based on an efficient in situ chelation and hard induction strategy. The MoO_x_ nanoparticles anchor on the surface of the 3D N-doped carbon with sizes between 1.5 and 3.5 nm. Therefore, the volume change during charge and discharge can be effectively mitigated, while the three-dimensional carbon skeleton can provide a conductive network, thus improving the lithium storage performance (delivering specific capacities of 742 mA h g^−1^ at current density of 100 mA g^−1^ and 431 mA h g^−1^ at 1000 mA g^−1^ after 1000 cycles, respectively).

Satyanarayana’s group [66] used a facile high-energy ball-milling process followed by ultrasonication method to prepare MoO_3_/rGO composites. Same as above, the composites exhibit better electrochemical performance than just nanostructured due to the increase in surface area and conductivity

Although the specific capacities of three samples drop gradually in the first five cycles, the MoO_3_-10 wt % rGO sample still shows higher reversible capacity (568 mA h g^−1^ at a high current density of 500 mA g^−1^ even after 100 cycles) than commercially available graphite (Figure 4h) and good rate performance with specific capacity 502 mA h g^−1^ even at a higher current density of 1000 mA g^−1^, and it retained the specific capacity of 853 mA h g^−1^ as the current density switched from 1000 mA g^−1^ to 100 mA g^−1^ (Figure 4i). It is conceivable that, in addition to the theoretical capacity of the material itself, enhancing the conductivity of the materials and constructing hetero-structure composite materials is very effective at improving the actual capacities and cycling stability of the electrode materials.

## 5. Metal and Non-Metal-Doped Composite

As previously mentioned, metal doping molybdenum oxide can change the crystal structures and phases of molybdenum oxides. If the formed metal doping molybdenum oxides have a layered crystal structure, the interlamellar spacing will increase to a certain extent. On the other hand, the crystal structure of some metal doping molybdenum oxides, such as Li_2_MoO_4_, is NASICON-type, which can also provide an ion storage property. Meanwhile, carbon or nitrogen doping plays a typical role in modifying materials by enhancing the electron conductivity and introducing some active sites to improve the electrochemical performance of electrode materials for ion batteries or supercapacitors. Therefore, combining the metal doping and non-metal doping has been an attractive method recently. Hu and coworkers [68] designed a carbon-coated and K-doped MoO_3_ composite prepared by hydrothermal treatment followed by a high-temperature annealing to solve the problems of low conductivity and irreversible structure change impediment. The pre-insertion of a layer of K^+^ as a backbone not only provides stability to the lamellar structure but also prevents Li^+^ from inserting the intralayer sites of the MoO_3_ crystals [69,70]. Therefore, the reduced resistance of the modified material facilitates a performance improvement. The as-prepared K_0.046_MoO_3_@C composites can deliver the specific capacities of 258 and 118 mA h g^−1^ at the current densities of 30 and 3000 mA g^−1^ over the potential range of 1.5–4.0 V (vs. Li^+^/Li), respectively, displaying an outstanding rate capability. When cycled at 1500 mA g^−1^, it can retain 83.9% of the initial capacity after 500 cycles. Meanwhile, the calculated value of Li-diffusion coefficient for KMC-3 is 2.06 × 10^−12^ cm^2^ s^−1^, showing a good Li^+^ diffusion efficiency.

**Figure 5:**
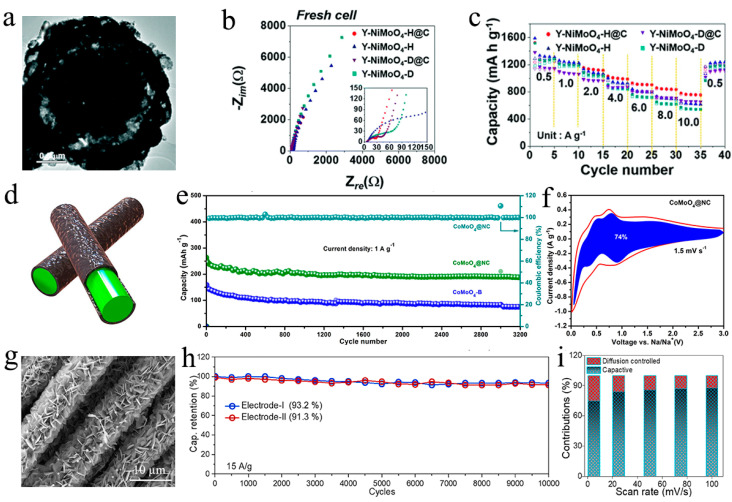
(**a**) TEM image of N-doped carbon coated hollow NiMoO_4_ nanospheres. (**b**) Nyquist plots of Y-NiMoO_4_-H@C, Y-NiMoO_4_-H, Y-NiMoO_4_-D, and Y-NiMoO_4_-D@C of fresh cells. (**c**) Rate performances of Y-NiMoO_4_-H@C. Reproduced with permission. [71] Copyright 2019, The Royal Society of Chemistry. (**d**) Schematic illustration of CoMoO_4_@NC nanorods; the green part is rod-like CoMoO_4_, and the coated part is N-doped carbon coatings. (**e**) Long-term cyclic performance at 1 A g^−1^ of CoMoO_4_@NC and CoMoO_4_-B. (**f**) The separation of the capacitive and diffusion currents of CoMoO_4_@NC at a scan rate of 1.5 mV s^−1^; the capacitive contribution to the total current is presented by the shaded region. Reproduced with permission. [73] Copyright 2020, Elsevier Inc. (**g**) SEM of CoMoO_4_@CFC (hexagonal nanosheets CoMoO_4_ are grown on the Carbon Fabric Cloth). (**h**) Cycling performance up to 10,000 cycles at current density of 15 A g^−1^ for two separate electrodes. (**i**) Capacitive and diffusive contribution at different scan rates. Reproduced with permission. [74] Copyright 2020, Elsevier Ltd. and Techna Group S.r.l.

Earlier, yolk–shell-structured microspheres composed of N-doped-carbon-coated NiMoO_4_ hollow nanospheres (Y-NiMoO_4_-H@C) synthesized via a spray pyrolysis process as anode for Li-ion battery was reported by Kang’s group [71]. The TEM is shown in Figure 5a, and it is clear that there is the cavity in a single nanosphere. They compared the properties of hollow and dance structure of nanospheres and the samples whether in coated carbon or not. The Y-NiMoO_4_-H@C microspheres have the largest BET surface area (55.7 m^2^ g^−1^) and show the lowest Rct value owing to the highly conductive N-doped carbon layers (Figure 5b). Obviously, yolk–shell-structured microspheres deliver great coulombic efficiency, cycling performance, and rate performance. The specific data of rate performance can be depicted in Figure 5c (final discharge capacities of 1267, 1221, 1120, 991, 907, 839, and 757 mA h g^−1^ at current densities of 0.5, 1, 2, 4, 6, 8, and 10 A g^−1^, respectively), indicating that Y-NiMoO_4_-H@C have fast charging and discharging processes at high current densities. Compared with the phase-pure β-NiMoO_4_ yolk–shell sphere (719 mA h g^−1^ at 4 A g^−1^ in rate performance) [72], N-doping and carbon coating have greatly improved the capacity of NiMoO_4_ by increasing the specific surface area and enhancing the electroconductivity.

Recently, Zhu’s group [73] designed N-doped carbon-encapsulated CoMoO_4_ (CoMoO_4_@NC) nanorods by a facile co-precipitation method to obtain a long-cycle-life anode material for sodium-ion battery. As shown in Figure 5d, the N-doped carbon shell acts as a cushion to adapt to gross volume changes during Na^+^ insertion/extraction while enhancing the electronic conductivity and activating the surface sites of CoMoO_4_. In fact, it shows a prolonged cycle life, especially at a high current density of 1 A g^−1^ (Figure 5e). Interestingly, the calculated capacitive current of CoMoO_4_@NC shows that 74% of the total charge contributions are quantified as capacitive (Figure 5f). Analogously, Hussain and coworkers [74] reported a CoMoO_4_/C composite by co-friendly hydrothermal method as a positive electrode for pseudocapacitors. Binder-free CoMoO_4_ hexagonal nanosheets (SEM is shown in Figure 5g) were directly grown on the surface of conductive carbon fabric cloth (CoMoO_4_@CFC), and the hexagonal-like 2D structure possesses mesoporous characteristics (BET = 68 m^2^ g^−1^) with abundant electroactive sites as the main body of charge storage. The electrochemical properties show that the material has excellent cycling stability as the capacitance retained up to 93.2% after up to 10,000 cycles, especially at a high current density of 15 A g^−1^ (Figure 5h). In addition, Figure 5i indicates the capacitive charge storage contributions at different scan rates and, accordingly, the CoMoO_4_@CFC electrode was attributed to a high capacitive charge value of 75% at 2.5 mV s^−1^. Using carbon-based materials as a coating layer or as a base frame material is an effective strategy for constructing new nanostructures of composite materials. At the same time, the materials with heterogeneous structure tend to have better cycling stability due to structural stability and almost having no volume expansion problem.

## 6. Summary and Outlook

In this review, we have attempted to chart the significant progress in hetero-element-doped molybdenum oxides for ion batteries and supercapacitors. The summarized series data and parameters of some hetero-element-doped molybdenum oxides are shown in Table 1. As can be seen from Table 1, the hetero-element-doped molybdenum oxide materials are mainly applied to the anode materials of lithium-ion batteries at present, and a small portion of them are used as the cathode materials in ion batteries and electrodes in supercapacitors. When they are applied to batteries, these materials generally have a voltage window of about 0~3.0 V. Among these hetero-element-doped molybdenum oxide materials, there is no way to make a specific comparison of different materials due to the inconsistency in the current densities and the number of cycles during the test, but the performances of these materials are much better than that of pure molybdenum oxide without doping. At the same time, the nanostructures of the materials also have a great influence on the performance. Therefore, in addition to the selection of doping elements, researchers also need to find a suitable synthesis method to obtain a nanostructure with a larger specific surface area and a small volume expansion effect.

Doping with different elements can change the crystal structure of molybdenum oxides (enlarge the interlamellar spacing or completely convert them to tunnel-type), introduce more active sites to increase the diffusion rate of the ions in the solid phase, increase the conductivity of the material, as well as make the material structure not easy to collapse and expand. These developments have greatly enriched molybdenum oxide-based materials and pave the way for possible design ideas of commercial materials. In addition, the changes in the complex structure and composition evolution during the electrochemical reactions and ion storage mechanisms of some molybdenum oxide-based materials are clarified, although many details of the electrochemical mechanisms on molybdenum oxide-based materials remain controversial and unclear. Except for designing different doped compounds and microstructures of hetero-element-doped molybdenum oxides to improve the capacity and cyclability for energy storage systems, how to reduce irrepressible capacity is an issue that needs attention. In many studies, it has been found that the initial charge capacity is very high, and the subsequent capacity will have a large attenuation and then remain within a certain range, indicating that the coulombic efficiency of the first cycle is very low, especially in nanostructure materials. Doping with hetero-elements and confirming the synthetic process usually involves the design of nanostructures of electrode materials. It is worth noting that irreversible capacity loss is crucial to real full cells, compromising the energy density and even the effect of nanostructuring. Therefore, using the current advanced technology to find ways to improve the coulombic efficiency of the first cycle is worthy of attention. The development of hetero-element-doped molybdenum oxide materials still faces great challenges, but notable progress has been made in the past years. Significant progress has been made regarding the material synthesis, improving electrochemical performances, and understanding the charge transport mechanism used in the energy storage systems.

In order to obtain materials with better performance, in addition to continuing to try different synthesis methods to obtain single metal-element-doped molybdenum oxides with different nanostructures, we could explore the synthesis of more bimetal-element-doped molybdenum oxide materials on the basis of the existing work [33,34], which will contribute to a significant expansion for molybdenum oxide-based materials. Based on the high capacity of different element-doped molybdenum oxide materials for lithium ions, most of the materials are now studied as anode materials for lithium-ion batteries. The active ions of other ion batteries, such as sodium-ion, potassium-ion, magnesium-ion, etc., have a larger radius than lithium-ion batteries. Therefore, hetero-element-doped molybdenum oxides may not meet the performance requirements as anodes for other ion batteries, but we could try to use them as cathode materials, which is a direction that has not been extensively studied. For non-metallic doping, there are a few cases that use materials that can be carbonized in nature as the substrate to obtain peculiar nanostructured composite materials. This is also a promising research idea; after all, nature is always worth learning from. There is no doubt that a basic understanding of the electrode structure, electrode/electrolyte interface, and charge storage mechanism, as well as a good study of the electron and ion transport in electrode/electrolytes through theoretical calculations, have important guiding significance for the development of molybdenum oxide-based materials.

## Figures and Tables

**Table 1:** Summary of the properties and applications of some hetero-element-doped molybdenum oxide materials.

Active Component		Application Fields	Specific Capacity (mA h g^−1^)	Cycles	Current Density (mA g^−1^)	Voltage Window (V)	Refs.
Alkali metal	Na_1/3_MoO_2_·H_2_O	Li/Na dual-ion battery (cathode)	650	500	50	0~3.0	[18]
	Li_2_MoO_4_@C	Li-ion battery (anode)	504	150	100	0.02~3.0	[75]
	Li_2_Mo_4_O_13_	Li-ion battery (anode)	768	50	106	0.1~2.5	[24]
	Lithiated MoO_3_ Nanobelts	Li-ion battery (cathode)	220	15	50	0.15~3.0	[76]
Non-metal	3D-MoO_x_@CN-700	Li-ion battery (anode)	431	1000	1000	0.01~3.0	[66]
	MoO_3_/NC	Li-ion battery (anode)	1250	60	410	0.01~3.0	[77]
	a-MM/NCc	Sodium-ion battery (anode)	1253	50	100	0.01~3.0	[78]
	MoOC/N-doped C	Li-ion battery (anode)	793	100	100	0.01~3.0	[79]
Transition metal or doped non-metal composites	MnMoO_4_@C	Li-ion battery (anode)	1050	200	100	0~3.0	[54]
	NiMoO_4_	Sodium-ion battery (anode)	245	100	50	0~3.0	[45]
	Cu_3_Mo_2_O_9_	Li-ion battery (anode)	632	200	100	0.01~3.0	[53]
	MnMoO_4_/CoMoO_4_	Supercapacitor	187 (F g^−1^)	1000	1000	0.1~0.4	[80]
	CoMoO_4_/AF-CNT	Sodium-ion battery (anode)	220	200	100	0.01~3.0	[81]
	ZnMoO_4_/rGO	Li-ion battery (anode)	632	100	100	0.01~3.0	[82]
